# Microeconomics of Metabolism: The Warburg Effect as Giffen Behaviour

**DOI:** 10.1007/s11538-021-00952-x

**Published:** 2021-10-31

**Authors:** Jumpei F. Yamagishi, Tetsuhiro S. Hatakeyama

**Affiliations:** Department of Basic Science, Graduate School of Arts and Sciences, The University of Tokyo, 3-8-1 Komaba, Meguro-ku, Tokyo 153-8902 Japan

**Keywords:** Metabolic systems, Overflow metabolism, Theory of consumer choice, Reverse Warburg effect

## Abstract

Metabolic behaviours of proliferating cells are often explained as a consequence of rational optimization of cellular growth rate, whereas microeconomics formulates consumption behaviours as optimization problems. Here, we pushed beyond the analogy to precisely map metabolism onto the theory of consumer choice. We thereby revealed the correspondence between long-standing mysteries in both fields: the Warburg effect, a seemingly wasteful but ubiquitous strategy where cells favour aerobic glycolysis over more energetically efficient oxidative phosphorylation, and Giffen behaviour, the unexpected consumer behaviour where a good is demanded more as its price rises. We identified the minimal, universal requirements for the Warburg effect: a trade-off between oxidative phosphorylation and aerobic glycolysis and complementarity, i.e. impossibility of substitution for different metabolites. Thus, various hypotheses for the Warburg effect are integrated into an identical optimization problem with the same universal structure. Besides, the correspondence between the Warburg effect and Giffen behaviour implies that oxidative phosphorylation is counter-intuitively stimulated when its efficiency is decreased by metabolic perturbations such as drug administration or mitochondrial dysfunction; the concept of Giffen behaviour bridges the Warburg effect and the reverse Warburg effect. This highlights that the application of microeconomics to metabolism can offer new predictions and paradigms for both biology and economics.

**Supplementary Information:**

The online version supplementary material available at 10.1007/s11538-021-00952-x.

## Introduction

Metabolic behaviours of proliferating cells can be often explained as a consequence of rational regulation to optimize cellular growth rate, as successfully predicted by biological theories such as flux balance analysis (FBA) (Bordbar et al. 2014; Edwards et al. 2001; Ibarra et al. 2002; Schuetz et al. 2012). In contrast, microeconomics describes the behaviour of individuals assumed to have perfect rationality to maximize their utility (Box 1). There is an apparent analogy between the two fields as optimization problems (Carlson and Taffs 2010; Shoval et al. 2012). We pushed beyond this analogy to precisely map metabolism onto the theory of consumer choice in microeconomics and examined the correspondence between the Warburg effect and Giffen behaviour.

The Warburg effect is a ubiquitous phenomenon where aerobic glycolysis is favoured over the more energetically efficient oxidative phosphorylation even in the presence of abundant oxygen. It is widely observed in fast-growing mammalian cells such as cancer cells (Vander Heiden et al. 2009), stem cells (Higuera et al. 2012) and immune cells (O’Neill et al. 2016) and is known as a therapeutic target in cancer (Poteet et al. 2013; da Veiga et al. 2019; Vander Heiden 2011). Even though the Warburg effect is seemingly wasteful, it arises by selection of cancer cells under harsh microenvironment conditions (Damaghi et al. 2021; Gatenby and Gillies 2004), which suggests that the Warburg effect is a consequence of optimization. Several competing hypotheses for this behaviour have been proposed, e.g. respiration requires larger solvent capacity (intracellular volume) because of the faster turnover of glycolytic enzymes (Vazquez 2017); respiration retards the cellular redox state (Dai et al. 2016); glycolysis with lactate secretion is more efficient in the production of NADPH (Vander Heiden et al. 2009). Similar switching from respiration to fermentation, termed overflow metabolism, is also ubiquitously observed in yeasts (De Deken 1966) and *E. coli* (Vemuri et al. 2006), and similar hypotheses have also been proposed (Vazquez 2017; Basan et al. 2015; Szenk et al. 2017; Niebel et al. 2019). Note here that respiration and (oxidative) fermentation in microbes correspond to oxidative phosphorylation and aerobic glycolysis in cancer cells, respectively. Nevertheless, no unified theory of this ubiquitous phenomenon has been developed.

In addition, recent studies reported that drug administration (Poteet et al. 2013; da Veiga et al. 2019), mitochondrial dysfunction (Demetrius et al. 2015), or intercellular metabolite exchange (Lee and Yoon 2015) stimulates respiration and inhibits aerobic glycolysis. Since such phenomenon is a reversal of the Warburg effect, it is called the reverse, reversed, or inverse Warburg effect; in particular, the behaviour due to intercellular metabolite exchange is often referred to as the reverse Warburg effect, while the terminology is not yet settled in the field. In the present paper, we refer to the phenomenon caused by drug administration or mitochondrial dysfunction as the drug-induced or mitochondrial-dysfunction-induced reverse Warburg effect. Interestingly, such drug responses are observed ubiquitously, e.g. in yeasts (Postma et al. 1989; Verduyn et al. 1992) and fungi (Gallmetzer and Burgstaller 2002). Despite such ubiquity, the relationship between the Warburg effect and the drug-induced or mitochondrial-dysfunction-induced reverse Warburg effect is still unclear, and the existing hypotheses for the Warburg effect cannot explain it.

Also, Giffen behaviour represents the counter-intuitive phenomenon in microeconomics, where the demand for a good increases when its price rises. This is in stark contrast to the general pattern of human economic activities and is often called Giffen’s paradox (Heijman and Mouche 2011). Even though Giffen goods were theoretically predicted more than a century ago and a few possible examples have been considered, their practical existence and mechanisms remain controversial (Heijman and Mouche 2011; Jensen and Miller 2008).

**Table 1:** Mapping between microeconomics and metabolism

Microeconomics	Metabolism
Utility	Growth rate
Income	Intake of nutrient
Goods	Metabolic pathways
Demand for goods	Allocation of nutrient
Price of goods	Inefficiency of metabolism
Complementarity	Stoichiometry

## Model and Results

**Fig. 1:**
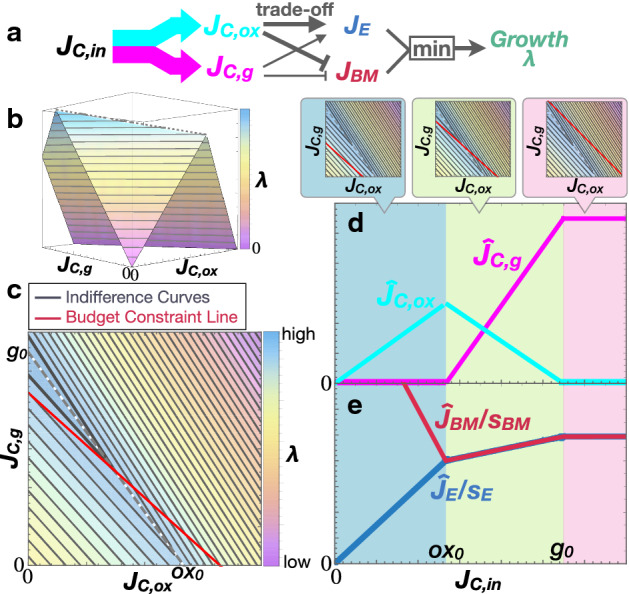
The Warburg effect as an optimization problem. **a** Schematic illustration of the microeconomics model. **b** Landscape of the growth rate $\lambda (J_{\mathrm{C}, \mathrm{ox}},J_{\mathrm{C}, \mathrm{g}})$. **c** Contour map of the growth rate. The indifference curves (contours of $\lambda$) and the budget constraint line (Eq. 1) are represented by black and red solid lines, respectively. The grey dashed line is the ridgeline of the growth rate (Eq. 5). The background colour represents the growth rate $\lambda (J_{\mathrm{C}, \mathrm{ox}},J_{\mathrm{C}, \mathrm{g}})$. **d** Dependence of the optimal allocation $(\hat{J}_{\mathrm{C}, \mathrm{ox}},\hat{J}_{\mathrm{C}, \mathrm{g}})$ on $J_{\mathrm{C},\mathrm{in}}$ (Engel curve; Eq. 15 in “Appendix 3”). **e** Dependence of $\hat{J}_\mathrm{E}/s_\mathrm{E}\equiv J_{E}(\hat{J}_{\mathrm{C}, \mathrm{ox}},\hat{J}_{\mathrm{C}, \mathrm{g}})/s_\mathrm{E}$ (blue line) and $\hat{J}_{\mathrm{BM}}/s_{\mathrm{BM}}\equiv J_{\mathrm{BM}}(\hat{J}_{\mathrm{C}, \mathrm{ox}},\hat{J}_{\mathrm{C}, \mathrm{g}})/s_{\mathrm{BM}}$ (dark-red line) on $J_{\mathrm{C},\mathrm{in}}$. The blue line also corresponds to the optimized growth rate $\lambda (\hat{J}_{\mathrm{C}, \mathrm{ox}},\hat{J}_{\mathrm{C}, \mathrm{g}})$. The top panels depict the contour maps for the cases $J_{\mathrm{C},\mathrm{in}}\le \mathrm {ox}_0$ (light-blue area), $g_0\ge J_{\mathrm{C},\mathrm{in}}\ge \mathrm {ox}_0$ (light-green area), and $J_{\mathrm{C},\mathrm{in}}\ge g_0$ (pink area) (Color figure online)

Here, we apply the theory of consumer choice to understand metabolic systems (Table 1). Our general theory shows that a trade-off is essential for the Warburg effect and integrates the aforementioned hypotheses into an identical optimization problem with the same universal structure (detailed in “Appendix 2”). In the main text, we explain only one of these for simplicity, i.e. a trade-off due to the allocation of limited resources other than nutrients. We consider a simple metabolic system that comprises the intake flux of the carbon source as a nutrient, $J_{\mathrm{C},\mathrm{in}}$, and fluxes to metabolize the nutrient to energy molecules in oxidative phosphorylation, $J_{\mathrm{C}, \mathrm{ox}}$, and glycolytic pathways, $J_{\mathrm{C}, \mathrm{g}}$ (Fig. 1a). $J_{\mathrm{C},\mathrm{in}}$ corresponds to the income, and $J_{\mathrm{C}, \mathrm{ox}}$ and $J_{\mathrm{C}, \mathrm{g}}$ correspond to the demand for goods. The budget constraint line is thus given by the carbon balance as:

1 $$\begin{aligned} J_{\mathrm{C}, \mathrm{in}}=p_{\mathrm{ox}} J_{\mathrm{C}, \mathrm{ox}}+p_\mathrm{g} J_{\mathrm{C}, \mathrm{g}}, \end{aligned}$$

which represents the allocation of nutrient intake to metabolic pathways.

In Eq. 1, $p_{\mathrm{ox}}$ and $p_\mathrm{g}$ are the “prices” of oxidative phosphorylation and aerobic glycolysis, respectively. Of note, when the price of some goods increases, consumers can get less amount of the goods from the same amount of money; in other words, the price in economics quantifies the inefficiency of conversion from money to goods. Thus, the price of a metabolic pathway is defined as the inverse of the efficiency to metabolize the nutrient, i.e. if the price of a metabolic pathway increases, only a smaller amount of the product will be obtained from an equal amount of the substrate. Moreover, as economic experiments control the prices of goods, the prices of pathways are defined as quantities controllable without any genetic manipulation. For example, if leakage or degradation of intermediate metabolites is increased by drugs, the price of the metabolic pathways increases. Without any loss of intermediates, the price is 1.

The cellular growth rate, $\lambda (J_{\mathrm{C}, \mathrm{ox}},J_{\mathrm{C}, \mathrm{g}})$, is the objective function (utility) for a cell. Because cells have to build their components from biomass precursors and energy molecules for successful division, $\lambda$ is determined from the production rates of energy molecules $J_\mathrm{E}(J_{\mathrm{C}, \mathrm{ox}},J_{\mathrm{C}, \mathrm{g}})$ and biomass precursors $J_{\mathrm{BM}}(J_{\mathrm{C}, \mathrm{ox}},J_{\mathrm{C}, \mathrm{g}})$, given as a function of the metabolic fluxes.

Here, each cellular component is built by metabolic reactions following the rules of stoichiometry. In general, due to the law of mass conservation, the compounds involved in biochemical reactions cannot be replaced by each other. Accordingly, the total amount of the product is determined by the least abundant component. This property of stoichiometry is identical to the concept of (perfect) complementarity in microeconomics, which is represented by a Leontief utility function, i.e. the least available element of all goods (Heijman and Mouche 2011). Hence, the growth rate is represented as:

2 $$\begin{aligned} \lambda (J_{\mathrm{C}, \mathrm{ox}},J_{\mathrm{C}, \mathrm{g}})=\min \left( \frac{1}{s_\mathrm{E}}J_{\mathrm{E}},\frac{1}{s_{\mathrm{BM}}}J_{\mathrm{BM}}\right) , \end{aligned}$$

where $s_\mathrm{E}$ and $s_{\mathrm{BM}}$ are the stoichiometric coefficients for energy molecules and biomass precursors to synthesize biomass, respectively.

The energy production flux, $J_{\mathrm{E}}$, is the sum of the fluxes of ATP synthesis via oxidative phosphorylation, $J_{\mathrm{E}, \mathrm{ox}}$, and aerobic glycolysis, $J_{\mathrm{E}, \mathrm{g}}$, which are proportional to $J_{\mathrm{C}, \mathrm{ox}}$ and $J_{\mathrm{C}, \mathrm{g}}$ with different coefficients, respectively:

3 $$\begin{aligned} J_{\mathrm{E}}\left( J_{\mathrm{C}, \mathrm{ox}},J_{\mathrm{C}, \mathrm{g}}\right) = J_{\mathrm{E}, \mathrm{ox}}+J_{\mathrm{E}, \mathrm{g}}=\epsilon _{\mathrm{ox}} J_{\mathrm{C}, \mathrm{ox}} + \epsilon _\mathrm{g} J_{\mathrm{C}, \mathrm{g}}, \end{aligned}$$

where $\epsilon _{\mathrm{ox}}$ and $\epsilon _\mathrm{g}$ are the efficiency of metabolism determined from stoichiometry and satisfy $\epsilon _{\mathrm{ox}}>\epsilon _\mathrm{g}>0$ because oxidative phosphorylation produces a greater amount of ATP than glycolysis from the same amount of the carbon source.

In contrast, by denoting the total amount of a given resource other than carbon, e.g., the intracellular space (Vazquez 2017), by $\rho _\mathrm{tot}$, the competition for the limited resource is described as $\rho _{\mathrm{ox}}+\rho _\mathrm{g}+\rho _{\mathrm{BM}}=\rho _\mathrm {tot}$, where $\rho _{\mathrm{ox}}$, $\rho _\mathrm{g}$, and $\rho _{\mathrm{BM}}$ are the resources allocated to oxidative phosphorylation, aerobic glycolysis, and production of biomass precursors, respectively. Based on the law of mass action, each flux is proportional to the allocated resource in the steady state: $J_{\mathrm{E}, \mathrm{ox}} = \epsilon ^\prime _{\mathrm{ox}} \rho _{\mathrm{ox}}$, $J_{\mathrm{E}, \mathrm{g}} = \epsilon ^\prime _\mathrm{g} \rho _\mathrm{g}$, and $J_{\mathrm{BM}} = \epsilon ^\prime _{\mathrm{BM}} \rho _{\mathrm{BM}}$. It follows that

4 $$\begin{aligned} J_{\mathrm{BM}}\left( J_{\mathrm{C}, \mathrm{ox}},J_{\mathrm{C}, \mathrm{g}}\right) =\epsilon ^\prime _{\mathrm{BM}}\rho _\mathrm {tot}-\epsilon ^\prime _{\mathrm{BM}}\frac{\epsilon _{\mathrm{ox}}}{\epsilon ^\prime _{\mathrm{ox}}} J_{\mathrm{C}, \mathrm{ox}}-\epsilon ^\prime _{\mathrm{BM}}\frac{\epsilon _\mathrm{g}}{\epsilon ^\prime _\mathrm{g}}J_{\mathrm{C}, \mathrm{g}}. \end{aligned}$$

Empirical observations show that oxidative phosphorylation requires more resources than glycolysis with lactate secretion, and thus, $\epsilon ^\prime _{\mathrm{ox}}<\epsilon ^\prime _\mathrm{g}$ holds (see Online Resource 1 for estimation of the parameters); note here that the higher speed in reactions is equivalent to the lower occupancy of some limited resources in the steady state (see also Szenk et al. 2017). Consequently, there is a trade-off for $J_\mathrm{E}(J_{\mathrm{C}, \mathrm{ox}},J_{\mathrm{C}, \mathrm{g}})$ and $J_{\mathrm{BM}}(J_{\mathrm{C}, \mathrm{ox}},J_{\mathrm{C}, \mathrm{g}})$ between $J_{\mathrm{C}, \mathrm{ox}}$ and $J_{\mathrm{C}, \mathrm{g}}$.

Under the above setting, the contours of the growth rate (indifference curves) are given as two-valued functions (Fig. 1b, c). Then, the growth rate is maximized at the tangent point of the budget constraint line (1) to the contour with the largest growth rate.

First, we show that the metabolic response against the carbon intake in the Warburg effect and overflow metabolism is explained as a result of optimization. Here, we discuss the properties of the optimized metabolic systems but do not discuss underlying mechanisms for optimization. We calculated the dependence of the optimal carbon allocation $(\hat{J}_{\mathrm{C}, \mathrm{ox}},\hat{J}_{\mathrm{C}, \mathrm{g}})$ on the income $J_{\mathrm{C},\mathrm{in}}$, termed the Engel curve in microeconomics, in the case without any drug, i.e. $p_{\mathrm{ox}}=p_\mathrm{g}=1$ (Fig. 1d). This Engel curve and the dependence of the optimal growth rate on $J_{\mathrm{C},\mathrm{in}}$ (Fig. 1d, e) are in good agreement with experimental observations (Vazquez et al. 2010; Vemuri et al. 2006; Basan et al. 2015; Vazquez 2017; Niebel et al. 2019).

If the influx of carbon sources $J_{\mathrm{C},\mathrm{in}}$ is lower than $\mathrm {ox}_0=\rho _\mathrm {tot}/(\frac{s_{\mathrm{BM}}\epsilon _{\mathrm{ox}}}{\epsilon ^\prime _{\mathrm{BM}}s_\mathrm{E}}+\frac{\epsilon _{\mathrm{ox}}}{\epsilon ^\prime _{\mathrm{ox}}})$, the budget constraint line has no tangent point to any contour, and the maximum growth rate is achieved at $(J_{\mathrm{C}, \mathrm{ox}}, J_{\mathrm{C}, \mathrm{g}}) = (J_{\mathrm{C},\mathrm{in}}, 0)$ (see the light-blue area in Fig. 1d, e). In this regime, the occupancy of limited resources $\rho _\mathrm {tot}$ does not limit cell growth, and all the carbon intake is used for oxidative phosphorylation to produce energy molecules more efficiently.

When the carbon intake $J_{\mathrm{C},\mathrm{in}}$ is sufficiently high, the budget constraint line has a tangent point to a contour. The set of such tangent points with various $J_{\mathrm{C},\mathrm{in}}$ is given as the line on which $\frac{1}{s_\mathrm{E}}J_{E}=\frac{1}{s_{\mathrm{BM}}}J_{\mathrm{BM}}$ (see the ridgeline in Fig. 1b, c, represented by the dashed lines):

5 $$\begin{aligned} J_{\mathrm{C}, \mathrm{g}}=-\frac{g_0}{\mathrm {ox}_0}J_{\mathrm{C}, \mathrm{ox}}+g_0, \end{aligned}$$

where $g_0 =\rho _\mathrm {tot}/(\frac{s_{\mathrm{BM}}\epsilon _\mathrm{g}}{\epsilon ^\prime _{\mathrm{BM}}s_\mathrm{E}}+\frac{\epsilon _\mathrm{g}}{\epsilon ^\prime _\mathrm{g}})$. Accordingly, the growth rate is maximized at the intersection between the above ridgeline (5) and the budget constraint line (1). Because the slope of the ridgeline (5) is negative due to the trade-off between the efficiency of energy production and resource occupancy, when $J_{\mathrm{C},\mathrm{in}}$ increases, the growth rate $\lambda (J_{\mathrm{C}, \mathrm{ox}},J_{\mathrm{C}, \mathrm{g}})$ and $\hat{J}_{\mathrm{C},\mathrm{g}}$ increase and $\hat{J}_{\mathrm{C}, \mathrm{ox}}$ decreases along the ridgeline (see the light-green area in Fig. 1d, e), i.e. the Warburg effect occurs. Also, a quantitative criterion of the Warburg effect $W\equiv \hat{J}_{\mathrm{C},\mathrm{g}}/\hat{J}_{\mathrm{C}, \mathrm{ox}}$, previously proposed in Dai et al. (2016), increases with $J_{\mathrm{C},\mathrm{in}}$.

From the viewpoint of microeconomics, the suppression of oxidative phosphorylation against the increase in $J_{\mathrm{C},\mathrm{in}}$ indicates the negative income effect of oxidative phosphorylation. Because the Leontief utility function has a null substitution effect (Heijman and Mouche 2011), the Warburg effect is immediately identified as Giffen behaviour where the oxidative phosphorylation pathway is a Giffen good (see also Box 1).

When $J_{\mathrm{C},\mathrm{in}}$ increases further and exceeds $g_0$, the optimal solution always takes the value $(\hat{J}_{\mathrm{C}, \mathrm{ox}}, \hat{J}_{\mathrm{C}, \mathrm{g}})=(0, g_0)$ (see the pink area in Fig. 1d, e) because the global maximum of the growth rate is achieved at this point. In this situation, the efficiency of producing energy molecules is no longer a primary concern given the excess carbon sources available, and the occupancy of the limited resources is the only selection criterion for the two metabolic pathways.

Remarkably, if there is no trade-off, i.e. both $\epsilon _{\mathrm{ox}}>\epsilon _\mathrm{g}$ and $\frac{\epsilon _{\mathrm{ox}}}{\epsilon ^\prime _{\mathrm{ox}}}<\frac{\epsilon _\mathrm{g}}{\epsilon ^\prime _\mathrm{g}}$ hold, the maximal point of the growth rate is not $(J_{\mathrm{C}, \mathrm{ox}}, J_{\mathrm{C}, \mathrm{g}}) = (0, g_0)$ but rather $(J_{\mathrm{C}, \mathrm{ox}}, J_{\mathrm{C}, \mathrm{g}}) =(\mathrm {ox}_0, 0)$. Then, cells allocate the carbon intake to only oxidative phosphorylation until reaching the upper bound determined by the available resources at $J_{\mathrm{C}, \mathrm{ox}}=\mathrm {ox}_0$, whereas $\hat{J}_{\mathrm{C},\mathrm{g}}$ remains at zero. A trade-off is thus required for the Warburg effect and also for Giffen behaviour.

**Fig. 2:**
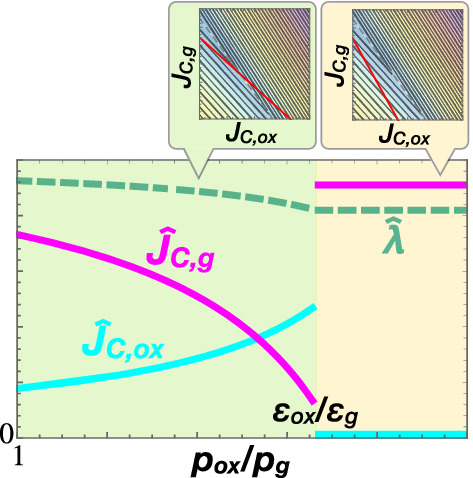
Dependence of the optimal allocation on price of oxidative phosphorylation $p_{\mathrm{ox}}$ (Eq. 16 in “Appendix 3”). $J_{\mathrm{C},\mathrm{in}}>\frac{\epsilon _{\mathrm{ox}}}{\epsilon _\mathrm{g}}\mathrm {ox}_0$ and $p_\mathrm{g}=1$ are fixed here. The cyan, magenta, and green curves depict $\hat{J}_{\mathrm{C}, \mathrm{ox}}$, $\hat{J}_{\mathrm{C}, \mathrm{g}}$, and $\hat{\lambda }\equiv \lambda (\hat{J}_{\mathrm{C}, \mathrm{ox}},\hat{J}_{\mathrm{C}, \mathrm{g}})$, respectively (scaled with different units). The top panels depict the contour maps for regimes (I) $p_{\mathrm{ox}}<\epsilon _{\mathrm{ox}}/\epsilon _\mathrm{g}$ (light-green area) and (II) $p_{\mathrm{ox}}>\epsilon _{\mathrm{ox}}/\epsilon _\mathrm{g}$ (yellow area) (Color figure online)

Based on the correspondence between the Warburg effect and Giffen behaviour, our theory can explain the mechanism of the drug-induced or mitochondrial-dysfunction-induced reverse Warburg effect (Demetrius et al. 2015; Poteet et al. 2013; da Veiga et al. 2019). In metabolism, when uncouplers of oxidative phosphorylation are administered, it becomes less efficient due to dissipation of the proton gradient to produce ATP (Brody 1955), i.e. the price of oxidative phosphorylation $p_{\mathrm{ox}}$ increases. Here, oxidative phosphorylation corresponds to a Giffen good, which is demanded more as its price rises; that is, the increases in $p_{\mathrm{ox}}$ will counterintuitively enhance the carbon flux towards oxidative phosphorylation with reduced efficiency (Fig. 2).

Of note, the property of optimal carbon allocation $(\hat{J}_{\mathrm{C}, \mathrm{ox}},\hat{J}_{\mathrm{C}, \mathrm{g}})$ qualitatively switches depending on whether the price ratio $p_{\mathrm{ox}}/p_\mathrm{g}$ is higher or lower than $\epsilon _{\mathrm{ox}}/\epsilon _\mathrm{g}$. In regime (I) $p_{\mathrm{ox}}/p_\mathrm{g}<\epsilon _{\mathrm{ox}}/\epsilon _\mathrm{g}$ (the light-green area in Fig. 2), the demand $\hat{J}_{\mathrm{C}, \mathrm{ox}}$ increases along with the price $p_{\mathrm{ox}}$, i.e. Giffen behaviour is observed. In contrast, in regime (II) $p_{\mathrm{ox}}/p_\mathrm{g}>\epsilon _{\mathrm{ox}}/\epsilon _\mathrm{g}$ (the yellow area in Fig. 2), using only glycolysis as $(J_{\mathrm{C}, \mathrm{ox}}, J_{\mathrm{C}, \mathrm{g}}) = (0, J_{\mathrm{C},\mathrm{in}}/p_\mathrm{g})$ is optimal in terms of growth rate. In this regime, considering the imbalance in price, the glycolytic pathway becomes more efficient for both $J_\mathrm{E}$ and $J_{\mathrm{BM}}$, and thus, there is no longer a trade-off (“Appendix 2”). Thus, the optimal allocation of carbon fluxes discontinuously jumps from regime (I) to (II) when $p_{\mathrm{ox}}$ increases, whereas the optimal growth rate changes continuously (Fig. 2). In addition, in the case of $\epsilon _{\mathrm{ox}} \mathrm {ox}_0 / \epsilon _\mathrm{g}>J_{\mathrm{C}, \mathrm{in}}>\mathrm {ox}_0$, $\hat{J}_{\mathrm{C},\mathrm{g}}$ reaches zero at $p_{\mathrm{ox}}/p_\mathrm{g}=J_{\mathrm{C},\mathrm{in}}/\mathrm {ox}_0$ and then $\hat{J}_{\mathrm{C}, \mathrm{ox}}$ continuously decreases before switching from regime (I) to (II) (Fig. S1 in Online Resource 1). In fact, in an yeast experiment, sudden reduction of oxidative phosphorylation and stimulation of aerobic glycolysis against addition of uncouplers above a critical concentration was observed (Verduyn et al. 1992).

## Discussion

Our study is based on the spirit inherited from constraint-based modelling that evolution forces metabolic systems to become optimized (Edwards et al. 2001; Bordbar et al. 2014; Schuetz et al. 2012), but we adopted a reductionist approach here. By constructing a simplified utility landscape comprising only a few variables with the aid of microeconomics, we uncovered the minimal, universal requirements for the Warburg effect and Giffen behaviour: trade-off and complementarity, i.e. impossibility of substitution for different goods. It no longer depends on the specific assumption of limited resource allocation (“Appendix 2”). Moreover, such extraction of the essence of the Warburg effect by microeconomic concepts offers a novel theory for the reverse Warburg effect induced by the uncoupler administration or mitochondrial dysfunction. Addition of uncouplers is also known to induce hyperthermia (Brody 1955; Vander Heiden 2011), and then, Giffen behaviour also connects drug-associated overheating with the Warburg effect by the same mechanism: drug administration increases the flux in the more dissipative and exothermic reactions.

In general, if increased intake of a substrate suppresses some flux, the flux will be increased by making the metabolic pathway less efficient. This is because perfect complementarity necessarily holds in metabolic systems, owing to the law of mass conservation, as complementarity represents the requirement of balance between metabolic fluxes. Notably, Giffen behaviour requires multiple pathways making the same product because an income effect must be non-negative if there is only one pathway. These results imply that a trade-off between different metabolic pathways immediately leads to Giffen behaviour because trade-offs cause the negative income effect. One representative example is the Embden–Meyerhof–Parnas (EMP) and Entner–Doudoroff (ED) pathways for the glycolytic strategy. The EMP pathway is more efficient for ATP production but requires a greater amount of enzymes than the ED pathway (Flamholz et al. 2013); accordingly, the EMP pathway is expected to behave as a Giffen good. Indeed, at high growth rates, the ED pathway is utilized to catabolize glucose (Flamholz et al. 2013). Other examples of trade-offs are as follows: a trade-off for ATP production and enzyme occupancy between the mixed-acid and lactic-acid fermentation pathways (Thomas et al. 1979); a trade-off for ATP synthesis and occupancy of the biomembrane between photosynthesis and glycolysis (Amthor 1995); trade-offs for the production of different metabolites from resources taken up by extracellular carbon, nitrogen, and phosphorus-acquiring enzymes (Sinsabaugh and Moorhead 1994); and a trade-off for carbon fixation and light harvesting in nitrogen allocation towards rubisco and chlorophyll (Henry and Aarssen 1997). Giffen behaviour should be ubiquitously observed in these metabolic systems. Furthermore, Giffen behaviour is interpretable as a novel mechanism of homeostasis because it seems to compensate for the loss of efficiency in some metabolic pathways by increasing its flux.

The optimal solutions on the ridgeline in our theory can reproduce the phenomenological model by Basan et al. (2015), though it is not explicitly formulated as an optimization problem and thus cannot systematically explain the metabolic strategies in response to drugs. Therefore, it could not address, for example, the link between the Warburg effect and the drug-induced or mitochondrial-dysfunction-induced reverse Warburg effect.

We also expect that the other metabolic behaviours characteristic to cancer will be understood by the microeconomics of metabolism: e.g. serine and glycine metabolism (Amelio et al. 2014; Meiser et al. 2018) and metabolic responses to drugs (Vander Heiden 2011). To understand multicellular behaviour of cancer cells, consideration of population dynamics is necessary. In the previous studies, the analogy with economics and ecology has been discussed in consumer-resource models (Lehman and Tilman 2000; Tilman et al. 2005); by introducing our novel approach of the microeconomics of metabolism to consumer-resource models (Amend et al. 2018; Orlando et al. 2012), we can investigate more complex phenomena in cancer, e.g. the reverse Warburg effect due to lactate exchange with stromal cells or other cancer cells (Lee and Yoon 2015) and the origin of cancer stem cells (Pacini and Borziani 2014). Note here that the Warburg effect is commonly characteristic of both cancer and stem cells.

From the perspective of microeconomics, we provided a concrete example of and a novel prediction for Giffen goods. The results are consistent with and uncovered a possible mechanism for an economic experiment in which Giffen behaviour is observed with moderate income, but cannot be observed in the case of extreme poverty (Jensen and Miller 2008). In our model, Giffen behaviour occurs in the intermediate range of income where the trade-off matters (Fig. 1), whereas, when the income is sufficiently low, only one objective affects the utility and a good then becomes the perfect substitute for the other one; in other words, the extremely poor cannot have multiple choices. Notably, perfect complementarity does not necessarily exist in Giffen behaviour outside of metabolism (see Online Resource 1).

We have paved the road for the field of microeconomics of metabolism, using the Warburg effect and Giffen behaviour as stepping stones. This will bring about further development in both biology and economics.
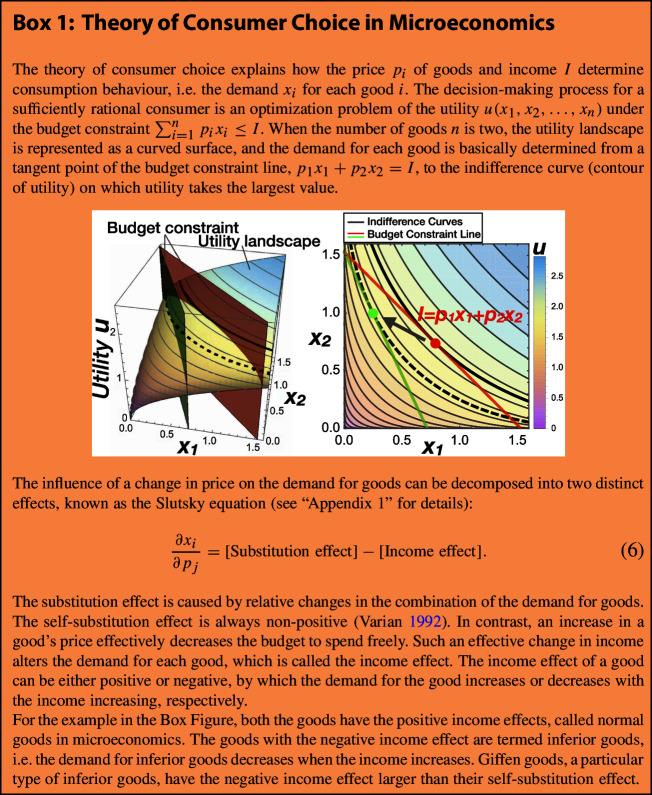


### Supplementary Information

Below is the link to the electronic supplementary material.

Supplementary material 1 (pdf 2366 KB)

## Figures and Tables

**Fig. 3:**
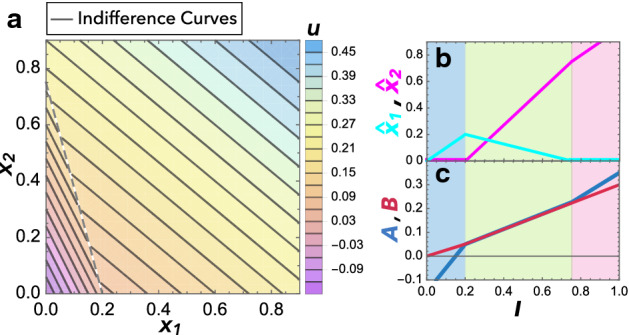
Example of a generalized model with the coefficients $a_1,a_2,b_1,b_2>0$. **a** Contour map. Indifference curves are shown as grey lines, and the dashed line depicts the ridgeline (10) on which $A(x_1,x_2)=B(x_1,x_2)$ holds. The background colour exhibits the value of the Leontief-type utility $u(x_1,x_2)=\min (A,B)=\min (a_1x_1+a_2x_2+A_0,b_1x_1+b_2x_2+B_0)$ (Eq. 9). **b** The Engel curve: $x_1$ (cyan line) and $x_2$ (magenta line) in the optimal solutions are plotted against the income *I*. **c** *A* (blue line) and *B* (dark-red line) in the optimal solutions are plotted against the income *I*. In (**b**–**c**), the price $p_1$ and $p_2$ are set at unity. In this example, the coefficients are set such that $a_1>a_2>b_2>b_1$ and $B_0>A_0$: $a_1=1,a_2=0.5,b_1 = 0.25,b_2 = 0.3,A_0=-0.15,B_0=0$. Since there is a trade-off between the production efficiencies of *A* and *B* and condition (11) is satisfied, Giffen behaviour is observed (Color figure online)

## Appendix 1: Substitution Effect and Income Effect

The influence of a change in price on the demand for goods can be decomposed into an income effect and a substitution effect as mentioned in the main text. This is known as Hicksian decomposition or the Slutsky equation (Varian 1992).

We here define $x_i(\mathbf{p} ,I)$ as the (optimal) demand for good *i* as a function of the price of goods $\mathbf{p}$ and income *I*. $E(\mathbf{p} ,u)$ represents the minimum income required for a given utility value *u* under price $\mathbf{p}$, and thus, $h_i(\mathbf{p} ,u)\equiv x_i(\mathbf{p} ,E(\mathbf{p} ,u))$ is the smallest demand for good *i* necessary to achieve the given utility value *u*. We can differentiate this with respect to $p_j$ and obtain

7 $$\begin{aligned} \frac{\partial h_i(\mathbf{p} ,\hat{u}(\mathbf{p} ,I))}{\partial p_j}= \frac{\partial x_i(\mathbf{p} ,I)}{\partial p_j}+ \frac{\partial x_i(\mathbf{p} ,I)}{\partial I}\frac{\partial E(\mathbf{p} ,\hat{u}(\mathbf{p} ,I))}{\partial p_j}, \end{aligned}$$

where $\hat{u}(\mathbf{p} ,I)$ is the maximal utility with the given price $\mathbf{p}$ and income *I*. Note that the last term, ${\partial E(\mathbf{p} ,{\hat{u}}(\mathbf{p} ,I))}/{\partial p_j}$, is simply equal to the demand $x_j$ under price $\mathbf{p}$ and income *I*.

Accordingly, the change in $x_i(\mathbf{p} ,I)$ due to a change in $p_j$ is given by the Slutsky equation:

8 $$\begin{aligned} \frac{\partial x_i(\mathbf{p} ,I)}{\partial p_j}= \frac{\partial h_i(\mathbf{p} ,\hat{u}(\mathbf{p} ,I))}{\partial p_j}-x_j(\mathbf{p} ,I)\frac{\partial x_i(\mathbf{p} ,I)}{\partial I}. \end{aligned}$$

The first term, ${\partial h_i(\mathbf{p} ,{\hat{u}})}/{\partial p_j}$, is the substitution effect, which is caused by relative changes in the combination of price values (Mankiw 2016). The case of $i=j$ represents the self-substitution effect, which is proven to be always non-positive (Varian 1992), i.e. the substitution effect never increases the demand for a good when its own price increases. In contrast, the second term, $x_j(\mathbf{p} ,I)\frac{\partial x_i(\mathbf{p} ,I)}{\partial I}$, is the income effect, which can be either positive or negative. This effect reflects the change in the demand for goods due to the effective decrease of income that is caused by increasing the price of a good. According to Eq. 8, these two effects determine the dependence of the demand for goods on the price (see also Table 2).

The substitution effect is represented as movement of the combination of the demand for goods along an indifference curve to a point at which the tangent line has a slope that is equal to the ratio of the altered price of goods. Hence, if the utility is given as a Leontief utility function, the substitution effect is zero within a certain range of the price change due to the indifferentiability of each indifference curve at the kink (Lancaster 1966). It follows that the substitution effect of the utility is always zero in the case of metabolic systems. Consequently, whether or not a metabolic pathway is a Giffen good depends only on the sign of its income effect; namely, a metabolic pathway behaves as a Giffen good if its income effect is negative. In the case of the Warburg effect and overflow metabolism, when the budget constraint line intersects with the ridgeline (Eq. 5), the income effect is negative for the utility $\lambda (J_{\mathrm{C}, \mathrm{ox}},J_{\mathrm{C},\mathrm{g}})$, and thus, Giffen behaviour is observed.

Note that although the demand for branded goods also increases with their price, they are not Giffen goods. This is because the demand for branded goods increases with the income, in other words, their income effect is positive. Such goods are called Veblen goods in microeconomics (Leibenstein 1950).

**Table 2:** Microeconomic properties of goods

Type of goods	Self-substitution effect	Income effect	Income $\uparrow$	Price $\uparrow$
Normal good	Non-positive	Positive	Demand $\uparrow$	Demand $\downarrow$
Inferior good	Non-positive	(Slightly) negative	Demand $\downarrow$	Demand $\downarrow$
Giffen good	Non-positive	Negative	Demand $\downarrow$	Demand $\uparrow$

## Appendix 2: Generalisation of the Theory of Consumer Choice for the Warburg Effect

The general theory for two goods and two complementary “objectives” elucidates the minimal requirements for Giffen behaviour, which is applicable to a variety of phenomena in biology and economics.

Let us define a Leontief utility function

9 $$\begin{aligned} u(x_1,x_2)\equiv \min (A,B), \end{aligned}$$

where two complementary objectives, *A* and *B*, are defined as

$$\begin{aligned} {\left\{ \begin{array}{ll} A(x_1,x_2) = a_1x_1+a_2x_2+A_0,\\ B(x_1,x_2) = b_1x_1+b_2x_2+B_0. \end{array}\right. } \end{aligned}$$

The demand for and price of two goods are represented by $x_1,x_2$ and $p_1,p_2$, respectively. The budget constraint line is thus $I=p_1x_1+p_2x_2$.

With respect to utility (9), the model proposed for the Warburg effect in the main text corresponds to the case where the signs of the parameters are given as $$\mathrm{sgn}\begin{pmatrix}a_1 &{} a_2 \\ b_1 &{} b_2\end{pmatrix}=\begin{pmatrix}+ &{} + \\ - &{} -\end{pmatrix}$$, while other sets of parameters also evoke the Warburg effect and Giffen behaviour. For instance, a trade-off between the efficiencies of producing different molecules such as ATP and NADPH (Vander Heiden et al. 2009) can also cause the Warburg effect, where the parameters are given by $$\mathrm{sgn}\begin{pmatrix}a_1 &{} a_2 \\ b_1 &{} b_2\end{pmatrix}=\begin{pmatrix}+ &{} + \\ + &{} +\end{pmatrix}$$ (Fig. 3). Indeed, the Leontief utility function with $$\mathrm{sgn} \begin{pmatrix}a_1 &{} a_2 \\ b_1 &{} b_2\end{pmatrix}=\begin{pmatrix}+ &{} + \\ + &{} 0\end{pmatrix}$$ was recently reported to demonstrate Giffen behaviour in the context of microeconomics (Jensen and Miller 2008; Heijman and Mouche 2011), which is a special case of the utility function (9).

In this section, we demonstrate that the optimization problem of the above utility (9) with every set of parameters will always be reduced to the optimization problem with an identical structure to that shown in the main text, as long as it shows Giffen behaviour. Hence, the two conditions, i.e. complementarity and trade-off, are required in all cases. Moreover, we expand the trade-off to include the effect of price.

First, we consider the simple case where $p_1 = p_2 = 1$. We here assume a trade-off between goods 1 and 2 as $a_1>a_2$ and $b_1<b_2$, whereas without the trade-off, the optimal strategy is to use either good 1 or 2 only. Owing to the trade-off, the utility is maximized on the ridgeline

10 $$\begin{aligned} x_2 = - \frac{a_1 - b_1}{a_2 - b_2} x_1 - \frac{A_0 - B_0}{a_2 - b_2} \end{aligned}$$

where $A(x_1,x_2)=B(x_1,x_2)$ holds. Giffen behaviour can be observed when this ridgeline has a negative slope and exists on the first quadrant, i.e. when $-(a_1-b_1)/(a_2-b_2)<0$ and $(B_0-A_0)/(a_2-b_2)>0$ hold.

From symmetry between $A(x_1,x_2)$ and $B(x_1,x_2)$, it is sufficient to consider the situation where

11 $$\begin{aligned} a_1>b_1,\quad a_2>b_2,\quad B_0>A_0 \end{aligned}$$

are satisfied. Note that this is the condition for a comparative advantage (Ricardo 1817).

If both $b_1$ and $b_2$ are non-positive and $a_1$ and $a_2$ are positive, condition (11) is autonomously satisfied. Then, the income effect is negative and Giffen behaviour is observed, as shown in the main text (see Fig. 1).

Even if $b_1$ or $b_2$ is positive, condition (11) can be satisfied as long as the condition $\mathrm{sgn}(a_1 - b_1) = \mathrm{sgn}(a_2 - b_2)$ is satisfied, as in Fig. 3. As discussed below, even such cases can be reduced to an optimization problem with the same universal structure demonstrated in the main text.

When Giffen behaviour can occur (i.e. condition (11) is satisfied), we can take a real number *c* such that $a_2>c>b_2$, and utility (9) can be represented as:

12 $$\begin{aligned} u(x_1,x_2)= & {} \min (A,B) \nonumber \\= & {} c(x_1+x_2)+A_0 +\, \min \Bigg ((a_1-c)x_1+(a_2-c)x_2, (b_1-c)x_1\nonumber \\&\quad +(b_2-c)x_2+B_0-A_0\Bigg ) \nonumber \\= & {} cI+A_0+\min (A^\prime ,B^\prime ), \end{aligned}$$

where

$$\begin{aligned} {\left\{ \begin{array}{ll} A^\prime (x_1,x_2) = (a_1-c)x_1+(a_2-c)x_2 = a^\prime _1x_1+a^\prime _2x_2,\\ B^\prime (x_1,x_2) = (b_1-c)x_1+(b_2-c)x_2+B_0-A_0 = b^\prime _1x_1+b^\prime _2x_2+B^\prime _0. \end{array}\right. } \end{aligned}$$

Here, $a^\prime _1=a_1-c>a_2-c=a^\prime _2$, $b^\prime _1=b_1-c<b_2-c=b^\prime _2<0$, and $B^\prime _0 = B_0 - A_0 > 0$ are satisfied from condition (11). Because only the last term, $\min (A^\prime ,B^\prime )$, depends on allocation of the income to goods, the generalized model is reduced to the same optimization problem as proposed in the main text as long as it shows Giffen behaviour.

A similar argument remains valid even when considering changes in price, although in this case the definition of trade-offs needs to be expanded to include the effect of price. In this case, a real number *c* can be taken such that $a_2/p_2>c>b_2/p_2$. Then, utility (9) can be represented as:

13 $$\begin{aligned} u(x_1,x_2)= & {} c(p_1x_1+p_2x_2)+A_0 +\, \min \Bigg ((a_1-cp_1)x_1+(a_2-cp_2)x_2,(b_1-cp_1)\nonumber \\&\quad \times x_1+(b_2-cp_2)x_2+B_0-A_0\Bigg ) \nonumber \\= & {} cI+A_0+\min (A^\prime ,B^\prime ), \end{aligned}$$

where

$$\begin{aligned} {\left\{ \begin{array}{ll} A^\prime (x_1,x_2) = (a_1-cp_1)x_1+(a_2-cp_2)x_2 = a^\prime _1x_1+a^\prime _2x_2,\\ B^\prime (x_1,x_2) = (b_1-cp_1)x_1+(b_2-cp_2)x_2+B_0-A_0 = b^\prime _1x_1+b^\prime _2x_2+B^\prime _0. \end{array}\right. } \end{aligned}$$

If $a_1/a_2>p_1/p_2>b_1/b_2$ holds, $a^\prime _1=a_1-cp_1>a_2-cp_2=a^\prime _2$, $b^\prime _1=b_1-cp_1<b_2-cp_2=b^\prime _2<0$, and $B^\prime _0 = B_0 - A_0>0$ are satisfied. Again, the generalized models showing Giffen behaviour are reduced to the optimization problem with the same universal structure as demonstrated in the main text.

Of note, if the difference of the price between goods 1 and 2 is so large that $p_1/p_2$ is larger than $a_1/a_2$ or smaller than $b_1/b_2$, Giffen behaviour disappears even when condition (11) and the trade-off of efficiencies to produce *A* and *B* (i.e. $a_1>a_2$ and $b_1<b_2$) hold. In this case, we need to consider a trade-off including the price. If $p_1/p_2$ is larger than $a_1/a_2$, a unit of demand for good 2 produces more *A* than that for good 1 even when $a_1$ is higher than $a_2$. That is, there is no longer a trade-off between the production of *A* and *B*. Hence, the condition for a trade-off is rewritten as $a_1/p_1 > a_2 /p_2$ and $b_1 / p_1 < b_2 / p_2$ (or $a_1/p_1 < a_2 /p_2$ and $b_1 / p_1 > b_2 / p_2$).

Intuitively, the trade-off including the price corresponds to rescaling of the utility landscape so that the slope of the budget constraint line, $-\frac{p_1}{p_2}$, becomes equal to $-1$. If there is a trade-off between *A* and *B* in the rescaled landscape (i.e. $a_1/p_1 > a_2 /p_2$ and $b_1 / p_1 < b_2 / p_2$, or $a_1/p_1 < a_2 /p_2$ and $b_1 / p_1 > b_2 / p_2$), Giffen behaviour can be observed.

## Appendix 3: Dependence of the Optimal Strategy on the Income and Price

The contour of the growth rate $\lambda (J_{\mathrm{C}, \mathrm{ox}},J_{\mathrm{C}, \mathrm{g}})$ with the value $\tilde{\lambda }$, (i.e. indifference curve in microeconomics) is given as a two-valued function, as shown in Fig. 1c:

14 $$\begin{aligned} J_{\mathrm{C}, \mathrm{g}}={\left\{ \begin{array}{ll} -\frac{\epsilon _{\mathrm{ox}}}{\epsilon _\mathrm{g}}J_{\mathrm{C}, \mathrm{ox}}+\frac{s_\mathrm{E}}{\epsilon _\mathrm{g}}\tilde{\lambda }\quad \mathrm {if}\;\frac{1}{s_\mathrm{E}}J_\mathrm{E}\le \frac{1}{s_{\mathrm{BM}}}J_{\mathrm{BM}}\\ -\frac{\epsilon _{\mathrm{ox}}}{\epsilon _\mathrm{g}}\frac{\epsilon ^\prime _\mathrm{g}}{\epsilon ^\prime _{\mathrm{ox}}}J_{\mathrm{C}, \mathrm{ox}}+\frac{\epsilon ^\prime _\mathrm{g}}{\epsilon _\mathrm{g}}\left( \rho _\mathrm {tot}-\frac{s_{\mathrm{BM}}}{\epsilon ^\prime _{\mathrm{BM}}}\tilde{\lambda }\right) \quad \mathrm {if}\;\frac{1}{s_\mathrm{E}}J_\mathrm{E}\ge \frac{1}{s_{\mathrm{BM}}}J_{\mathrm{BM}} \end{array}\right. } \end{aligned}$$

The growth rate is maximized at the tangent point of the budget constraint line (Eq. 1) to the contour with the largest growth rate.

In the case of $p_{\mathrm{ox}}=p_\mathrm{g}=1$, the dependence of the optimal strategy $(\hat{J}_{\mathrm{C}, \mathrm{ox}},\hat{J}_{\mathrm{C},\mathrm{g}})$ on $J_{\mathrm{C},\mathrm{in}}$, called the Engel curve in microeconomics, is calculated (see also Fig. 1c):

15 $$\begin{aligned} \left( \hat{J}_{\mathrm{C}, \mathrm{ox}},\hat{J}_{\mathrm{C},\mathrm{g}}\right) ={\left\{ \begin{array}{ll} \left( J_{\mathrm{C},\mathrm{in}},0\right) ,\quad \mathrm {if}\;J_{\mathrm{C},\mathrm{in}}\le \mathrm {ox}_0\\ \left( \frac{\mathrm {ox}_0}{g_0-\mathrm {ox}_0}(g_0-J_{\mathrm{C},\mathrm{in}}),\frac{g_0}{g_0-\mathrm {ox}_0}(J_{\mathrm{C},\mathrm{in}}-\mathrm {ox}_0)\right) \\ \quad \quad \quad \quad \quad \quad \quad \quad \quad \quad \quad \quad \quad \quad \mathrm {if}\; g_0\ge J_{\mathrm{C},\mathrm{in}}\ge \mathrm {ox}_0\\ \left( 0,g_0\right) \quad \mathrm {if}\;J_{\mathrm{C},\mathrm{in}}\ge g_0 \end{array}\right. } \end{aligned}$$

Changes in price alter the demand for goods, and thus, the optimal strategy depends on the price $p_{\mathrm{ox}}$ and $p_\mathrm{g}$ as well as on the income $J_{\mathrm{C},\mathrm{in}}$. Accordingly, if the price $p_{\mathrm{ox}}$ or $p_\mathrm{g}$ takes a value larger than 1, the Engel curve is generalized as follows.

16 $$\begin{aligned} \left( \hat{J}_{\mathrm{C}, \mathrm{ox}},\hat{J}_{\mathrm{C},\mathrm{g}}\right) ={\left\{ \begin{array}{ll} \left( J_{\mathrm{C},\mathrm{in}}/p_{\mathrm{ox}},0\right) \quad \mathrm {if}\;J_{\mathrm{C},\mathrm{in}}\le p_{\mathrm{ox}}\mathrm {ox}_0\\ \left( \left( g_0-\frac{J_{\mathrm{C},\mathrm{in}}}{p_\mathrm{g}}\right) \bigg / \left( \frac{g_0}{\mathrm {ox}_0}-\frac{p_{\mathrm{ox}}}{p_\mathrm{g}}\right) ,\left( \frac{J_{\mathrm{C},\mathrm{in}}}{p_{\mathrm{ox}}}-\mathrm {ox}_0\right) \bigg /\left( \frac{p_\mathrm{g}}{p_{\mathrm{ox}}}-\frac{\mathrm {ox}_0}{g_0}\right) \right) \\ \quad \quad \quad \quad \quad \quad \quad \quad \quad \quad \quad \quad \quad \quad \quad \quad \mathrm {if}\; p_\mathrm{g}g_0\ge J_{\mathrm{C},\mathrm{in}}\ge p_{\mathrm{ox}}\mathrm {ox}_0\\ \left( 0,g_0\right) \quad \mathrm {if}\;J_{\mathrm{C},\mathrm{in}}\ge p_\mathrm{g}g_0 \end{array}\right. } \end{aligned}$$

with $\mathrm {ox}_0=\rho _\mathrm {tot}/(\frac{s_{\mathrm{BM}}\epsilon _{\mathrm{ox}}}{\epsilon ^\prime _{\mathrm{BM}}s_\mathrm{E}}+\frac{\epsilon _{\mathrm{ox}}}{\epsilon ^\prime _{\mathrm{ox}}})$ and $g_0=\rho _\mathrm {tot}/(\frac{s_{\mathrm{BM}}\epsilon _\mathrm{g}}{\epsilon ^\prime _{\mathrm{BM}}s_\mathrm{E}}+\frac{\epsilon _\mathrm{g}}{\epsilon ^\prime _\mathrm{g}})$. Here, the optimal $\left( J_{\mathrm{C}, \mathrm{ox}},J_{\mathrm{C},\mathrm{g}}\right)$ for the case $p_\mathrm{g}g_0\ge J_{\mathrm{C},\mathrm{in}}\ge p_{\mathrm{ox}}\mathrm {ox}_0$ is obtained from the intersecting point of the budget constraint line with the ridgeline (Eq. 5). Note that Eq. 16 is identical to Eq. 15 if $p_{\mathrm{ox}}=p_\mathrm{g}=1$ is satisfied.
