# Supplementary material for: Microeconomics of Metabolism: The Warburg Effect as Giffen Behaviour
Source: Bull Math Biol. 2021 Oct 31;83(12):120. doi: 10.1007/s11538-021-00952-x (PMC8558188; doi:10.1007/s11538-021-00952-x)
Supplement: Supplementary file 1 — Supplementary material 1 (pdf 2366 KB) [file 11538_2021_952_MOESM1_ESM.pdf]

S1. Estimation of parameters

In Figs. 2 and 3 in the main text, we used an arbitrarily chosen set of parameters ($\epsilon_{ox} = 0.75, \epsilon_g = 0.45, \epsilon'_{ox} = 0.5, \epsilon'_g = 0.7, \rho_{tot} = 0.6, \epsilon'_{BM} = 1, s_E = s_{BM} = 0.5$) in order to demonstrate a mathematical structure of the landscape clearly (see also Table S1 for meaning of the symbols). However, our results do not depend on the precise values of the parameters as long as the relative values of parameters satisfy the conditions described in the main text: $\epsilon_{ox} > \epsilon_g$ and $\frac{\epsilon_{ox}}{\epsilon'_{ox}} > \frac{\epsilon_g}{\epsilon'_g}$.

One can estimate the parameter set in actual cells and show that the estimated parameters indeed satisfy the conditions for overflow metabolism. The details are given below (summarized in Table S2).

First, the intake rate of the carbon source (e.g., glucose) is the order of 1 [mMol glucose/gDW] (1).

According to stoichiometry, respiration (i.e., full oxidation of glucose) can generate 36 ATP molecules per 1 molecule of glucose at most, while in reality it generates about $30 - 32$ ATP molecules per 1 molecule of glucose (2, 3). In contrast, aerobic glycolysis generates 2 ATP and 4 NADH, in total, from 1 glucose, and fermentation generates 2 ATP more. Besides, in the aerobic environment, 1 NADH molecule can be converted into, at most, 2.5 ATP molecules; thus, at most, 12 or 14 ATP molecules can be generated from 1 glucose by aerobic glycolysis or fermentation, respectively (2, 4). Then, the values of ϵ_{ox} and ϵ_g are dependent on the organism in question, but a requirement for overflow metabolism, $\epsilon_{ox} > \epsilon_g$, is always satisfied: $\epsilon_{ox} \simeq 32$ [mMol ATP/ mMol glucose] and $\epsilon_g = 2 \sim 12$ [mMol ATP/ mMol glucose]. Notably, the decrease in the optimal flux $\hat{J}_{C,ox}$ against nutrient supply $J_{C,in}$ can be very gentle (whereas glycolysis $\hat{J}_{C,g}$ increases steeply), e.g., in the situation where ϵ_{ox}/ϵ_g is relatively large (i.e., NADH produced in glycolysis is not converted to ATP consuming oxygen), as experimentally observed (3, 5).

The values and units of ρ_{tot}, ϵ'_{ox}, ϵ'_g, and s_{BM}/ϵ'_{BM} depend on the nature of the limited resource in question. When the limited resource ρ_{tot} is the total volume of mitochondria or solvent capacity of mitochondria in each cell such as cancer cells, muscle cells, and yeasts, the intracellular volume for proteins per cellular dry weight is $\rho_{tot} \simeq 3 \times 10^{-3}$ [L/gDW] and the approximate values of the other parameters are $\epsilon'_{ox} \simeq 3 \times 10^3$ [mMol ATP/L/hour], $\epsilon'_g \simeq 9 \times 10^4$ [mMol ATP/L/hour], and $s_{BM}/\epsilon'_{BM} \simeq 10^{-3}$ [hour × L/gDW] (3, 6).

Alternatively, when ρ_{tot} is the fraction of enzymes for growth in *E. coli*, the approximate values of the parameters are estimated as $\rho_{tot} \simeq 2 \times 10^{-1}$ [$A_{600\ \mathrm{nm}}$], $\epsilon'_{ox} \simeq 4 \times 10^2$ [mMol ATP/$A_{600\ \mathrm{nm}}$/hour] and $\epsilon'_g \simeq 8 \times 10^2$ [mMol ATP/$A_{600\ \mathrm{nm}}$/hour], and $s_{BM}/\epsilon'_{BM} \simeq 10^{-1}$ [hour] (4).

When the balance between cellular redox state and energy demand is considered (7), ρ_{tot} is the maximal flux of NAD^+ produced by the other cellular processes and is estimated as $\rho_{tot} \simeq 3 \times 10^2$ [mMol NAD^+/gWW/hour]. Then, $\rho_g = J_{E,g}/\epsilon'_g$ is 0 (i.e., ϵ'_g can be regarded as infinity) because glycolysis does not consume NAD^+. In contrast, respiration consumes 1 NAD^+ per 1 glucose, or per ϵ_{ox} ATP molecules; thus, the value of ϵ'_{ox} equals that of ϵ_{ox}, i,e., $\epsilon'_{ox} \simeq 32$ [mMol ATP/mMol NAD^+]. In addition, the amount of required NAD^+ per cellular biomass is estimated as $s_{BM}/\epsilon'_{BM} \simeq 2 \times 10^2$ [mMol NAD^+/gWW].

Because $\epsilon'_{ox} < \epsilon'_g$ holds in all the above cases, the other requirement for the Warburg effect and overflow metabolism, $\frac{\epsilon_{ox}}{\epsilon'_{ox}} > \frac{\epsilon_g}{\epsilon'_g}$, is also satisfied.

Finally, p_{ox} and p_g approximately equal 1 without the administration of drugs because, at most, only $\sim 10\%$ carbon is usually leaked as intermediates of central metabolism (8). In contrast, under the existence of drug such as uncouplers of respiration, p_{ox} is substantially larger than 1, quantified as the inverse of the ATP yield, i.e., the fraction of the non-dissipated proton.

S2. Giffen behaviour requires complementarity but not perfect complementarity

Based on biological considerations, we introduced the Leontief utility function $\lambda(J_{C,ox}, J_{C,g})$ that has perfect complementarity in the main text. However, Giffen behaviour can be observed for utility functions with only partial complementarity, as long as the substitution effect is sufficiently small.

An example of such utility functions is

$$u(x_1, x_2) \equiv \left[s_E / \left(\epsilon_{ox} x_1 + \epsilon_g x_2\right) + s_{BM} / \left(\rho_{\mathrm{tot}} - \frac{\epsilon_{ox}}{\epsilon'_{ox}} x_1 - \frac{\epsilon_g}{\epsilon'_g} x_2 \right) \epsilon'_{BM} \right]^{-1}. \quad \text{[S1]}$$

The landscape of this utility function (Fig. S2A) is similar to that of $\lambda(J_{C,ox}, J_{C,g})$ (Fig. 1a in the main text). As shown in Fig. S2B, x_1 with the utility [S1] showing Giffen behaviour within a range of the price of the good 1, p_1.

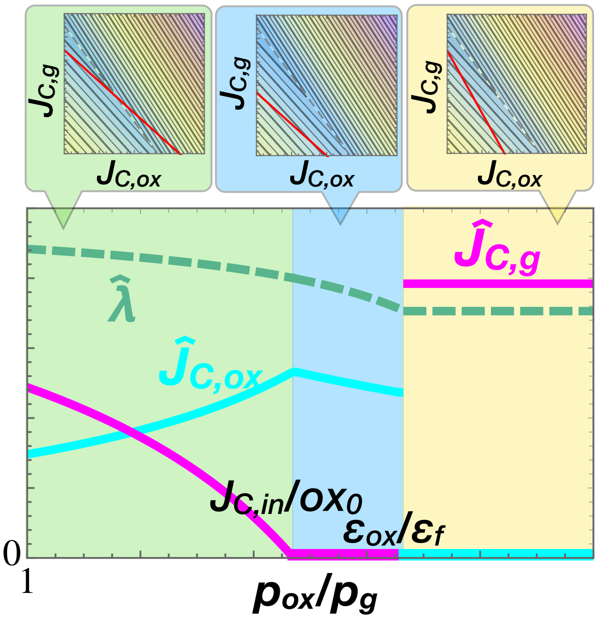

Figure S1. Dependence of the optimal strategy $(\hat{J}_{C,ox}, \hat{J}_{C,g})$ on the price of oxidative phosphorylation p_{ox}, where $J_{C,in} < \frac{\epsilon_{ox}}{\epsilon_g}\mathrm{ox}_0$, $J_{C,in} > \mathrm{ox}_0$, and $p_g = 1$ hold. The cyan, magenta, and green curves depict $\hat{J}_{C,ox}$, $\hat{J}_{C,g}$, and $\hat{\lambda} \equiv \lambda(\hat{J}_{C,ox}, \hat{J}_{C,g})$, respectively (scaled with different units). Top panels depict the contour maps and the budget constraint lines for regime (I) $p_{ox} < \epsilon_{ox}/\epsilon_g$ (light-green area) and $J_{C,in} \leq p_{ox}\mathrm{ox}_0$ and $p_{ox} < \epsilon_{ox}/\epsilon_g$ (light-blue area) and (II) $p_{ox} > \epsilon_{ox}/\epsilon_g$ (yellow area).

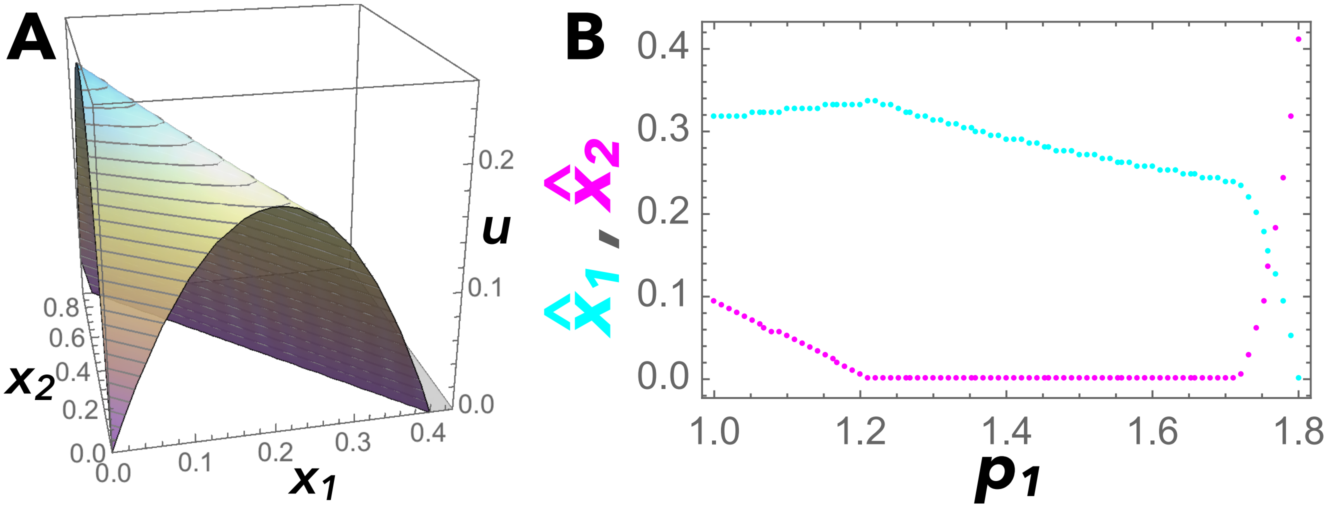

Figure S2. Example of utility functions showing Giffen behaviour without perfect complementarity. The utility function $u(x_1, x_2)$ is given by Eq. [S1]. (A) Utility landscape of $u(x_1, x_2)$ and (B) demand curves for goods 1 (light-blue dots) and 2 (pink dots). The optimal strategies $(\hat{x}_1, \hat{x}_2)$ were numerically calculated with the parameters $\epsilon_{ox} = 0.75, \epsilon_g = 0.4, \epsilon'_{ox} = 0.5, \epsilon'_g = 1, J_{C,in} = 0.4$; all other parameters (i.e., ρ_{tot}, ϵ'_{BM}, s_E, s_{BM}, and p_2) were set at unity.

Table S1. Biological and economic meanings of symbols.

Symbol	Biological meaning	Economic meaning
$J_{C,in}$	Intake flux of carbon source	Income
$J_{C,ox}, J_{C,g}$	Fluxes of carbon in oxidative phosphorylation and glycolysis	Demand for goods
p_{ox}, p_g	Inefficiency of metabolism in oxidative phosphorylation and glycolysis	Price of goods
λ	Growth rate	Utility
J_E	Total flux of ATP production	An objective
$J_{E,ox}, J_{E,g}$	Flux of ATP production by oxidative phosphorylation and glycolysis	
J_{BM}	Total flux of biomass precursors production	Another objective
ρ_{tot}	Total amount of the limited resource	
$\rho_{ox}, \rho_g, \rho_{BM}$	Fraction of the limited resource used for oxidative phosphorylation, glycolysis, and biomass synthesis	
$\epsilon_{ox}, \epsilon_g$	Stoichiometric efficiency of ATP production in oxidative phosphorylation and glycolysis	
$\epsilon'_{ox}, \epsilon'_g, \epsilon'_{BM}$	Occupancy rate of the limited resource for oxidative phosphorylation, glycolysis, and biomass synthesis	
s_E, s_{BM}	Stoichiometric constants	

Table S2. Approximate values of estimated parameters

Parameter	Value	Reference
ϵ_{ox}	~ 30 [mMol ATP/mMol glucose]	Refs. (2)
ϵ_g	$2 \sim 14$ [mMol ATP/mMol glucose]	Refs. (2)
p_{ox}	$\gtrsim 1$	Ref. (8)
p_g	~ 1	Ref. (8)
s_E	6×10^1 [mMol ATP/gDW]	Ref. (9)
Solvent capacity hypothesis		Ref. (3, 6)
ρ_{tot}	3×10^{-3} [L/gDW]	
ϵ'_{ox}	2×10^4 [mMol ATP/L/hour]	
ϵ'_g	9×10^4 [mMol ATP/L/hour]	
s_{BM}/ϵ'_{BM}	10^{-3} [hour × L/gDW]	
Proteome allocation hypothesis		Ref. (4)
ρ_{tot}	2×10^{-1} [$A_{600\ \mathrm{nm}}$]	
ϵ'_{ox}	4×10^2 [mMol ATP/$A_{600\ \mathrm{nm}}$/hour]	
ϵ'_g	8×10^2 [mMol ATP/$A_{600\ \mathrm{nm}}$/hour]	
s_{BM}/ϵ'_{BM}	10^{-1} [hour]	
Redox balance hypothesis		Ref. (7)
ρ_{tot}	3×10^2 [mMol NAD^+/gWW/hour]	
ϵ'_{ox}	3×10^1 [mMol ATP/mMol NAD^+]	
$\rho_g(\propto 1/\epsilon'_g)$	0 [mMol NAD^+/gWW/hour]	
s_{BM}/ϵ'_{BM}	2×10^2 [mMol NAD^+/gWW]	

Supplementary References

1. Shinfuku Y, et al. (2009) Development and experimental verification of a genome-scale metabolic model for Corynebacterium glutamicum. *Microb Cell Fact* 8:1–15.
2. Stryer, Lubert (1995). Biochemistry (fourth ed.). New York – Basingstoke: W. H. Freeman and Company.
3. Vazquez A, Liu J, Zhou Y, Oltvai ZN (2010) Catabolic efficiency of aerobic glycolysis: The Warburg effect revisited. *BMC Syst Biol* 4: 58.
4. Basan M, et al. (2015) Overflow metabolism in Escherichia coli results from efficient proteome allocation. *Nature* 528(7580):99–104.
5. Postma E, Verduyn C, Scheffers WA, Van Dijken JP (1989) Enzymic analysis of the crabtree effect in glucose-limited chemostat cultures of Saccharomyces cerevisiae. *Appl Environ Microbiol* 55(2):468–77.
6. Vazquez A (2017) Overflow Metabolism: From Yeast to Marathon Runners (Academic Press; Cambridge).
7. Dai Z, Shestov AA, Lai L, Locasale JW (2016) A Flux Balance of Glucose Metabolism Clarifies the Requirements of the Warburg Effect. *Biophys J* 111(5):1088-1100.
8. Paczia N, et al. (2012) Extensive exometabolome analysis reveals extended overflow metabolism in various microorganisms. *Microb Cell Fact* 11:1–14.
9. Szenk M, Dill KA, de Graff AMR (2017) Why Do Fast-Growing Bacteria Enter Overflow Metabolism? Testing the Membrane Real Estate Hypothesis. *Cell Syst* 5(2):95–104.
